# Evaluation of dried blood spot sampling for real-time PCR malaria diagnostics in a rural setting in Angola

**DOI:** 10.1186/s13071-025-06685-3

**Published:** 2025-02-07

**Authors:** Alejandro Mediavilla, Begoña Febrer-Sendra, Aroa Silgado, Patricia Martínez-Vallejo, Beatriz Crego-Vicente, Arlette Nindia, Carles Rubio Maturana, Lidia Goterris, Joan Martínez-Campreciós, Sandra Aixut, Pedro Fernández-Soto, María Luisa Aznar, Antonio Muro, Inés Oliveira-Souto, Israel Molina, Elena Sulleiro

**Affiliations:** 1 Microbiology Department, Vall d’Hebron University Hospital, Autonomous University of Barcelona, PROSICS Barcelona, Barcelona, Spain; 2 Department of Microbiology and Genetics, Universitat Autònoma de Barcelona (UAB), Barcelona, Spain; 3 Infectious and Tropical Diseases Research Group (E-INTRO), Biomedical Research Institute of Salamanca-Center for Research in Tropical Diseases of the University of Salamanca (IBSAL-CIETUS), Faculty of Pharmacy, University of Salamanca, Salamanca, Spain; 4 Centro de Investigación Biomédica en Red de Enfermedades Infecciosas (CIBERINFEC), Instituto de Salud Carlos III, Madrid, Spain; 5 Hospital Nossa Senhora da Paz, Cubal, Angola; 6 International Health Unit Vall d’Hebron-Drassanes, Infectious Diseases Department, Vall d’Hebron University Hospital, PROSICS Barcelona, Barcelona, Spain

**Keywords:** Malaria, Dried blood spots, Sampling, Real-time PCR, Diagnosis, Whole blood

## Abstract

**Background:**

Malaria is the parasitic disease with the highest morbidity and mortality worldwide. Angola is one of the five sub-Saharan African countries with the highest malaria burden. Real-time PCR diagnosis in endemic areas has not been implemented due to its high cost and the need for adequate infrastructure. Dried blood spots (DBSs) are an alternative for collecting, preserving, and transporting blood samples to reference laboratories. The objective of the study was to assess the efficacy of DBS as a sampling method for malaria research studies employing real-time PCR.

**Methods:**

The study was divided into two phases: (i) prospective study at the Hospital Universitario Vall d'Hebron (HUVH) to compare real-time PCR from whole blood or DBS, including 12 venous blood samples from patients with positive real-time PCR for *Plasmodium* spp. and 10 quality control samples (nine infected samples and one negative control). Samples were collected as DBSs (10, 20, 50 µl/circle). Samples from both phases of the study were analyzed by generic real-time PCR (*Plasmodium* spp.) and the subsequent positive samples underwent species-specific real-time PCR (*Plasmodium* species) and (ii) cross-sectional study conducted at the Hospital Nossa Senhora da Paz, Cubal (Angola), including 200 participants with fever. For each patient, a fresh capillary blood specimen [for thin and thick blood films and rapid diagnostic test (RDT)] and venous blood, collected as DBSs (two 10-µl circles were combined for a total volume of 20 µl of DBS), were obtained. DBSs were sent to HUVH, Barcelona, Spain.

**Results:**

(i) Real-time PCR from whole blood collection was positive for 100% of the 21 *Plasmodium* spp.-infected samples, whereas real-time PCR from DBSs detected *Plasmodium* spp. infection at lower proportions: 76.19% (16/21) for 10 µl, 85.71% (18/21) for 20 µl, 88.24% (15/17) for 50 µl and 85.71% (18/21) for 100 µl DBSs. (ii) Field diagnosis (microscopy and/or RDT) showed a 51.5% (103/200) positivity rate, while 50% (100/200) of the DBS samples tested positive by real-time PCR. Using field diagnosis as the reference method, the sensitivity of real-time PCR in DBS samples was 77.67% with a specificity of 79.38%. *Plasmodium* species were identified in 86 samples by real-time PCR: 81.40% (16/86) were caused by *Plasmodium falciparum*, 11.63% (10/86) were coinfections of *P. falciparum* + *P. malariae*, 4.65% (4/86) were *P. falciparum* + *P. ovale*, and 2.33% (2/86) were triple coinfections.

**Conclusions:**

The DBS volume used for DNA extraction is a determining factor in the performance of real-time PCR for *Plasmodium* DNA detection. A DBS volume of 50–100 µl appears to be optimal for malaria diagnosis and *Plasmodium* species determination by real-time PCR. DBS is a suitable method for sample collection in Cubal followed by real-time PCR analysis in a reference laboratory.

**Graphical Abstract:**

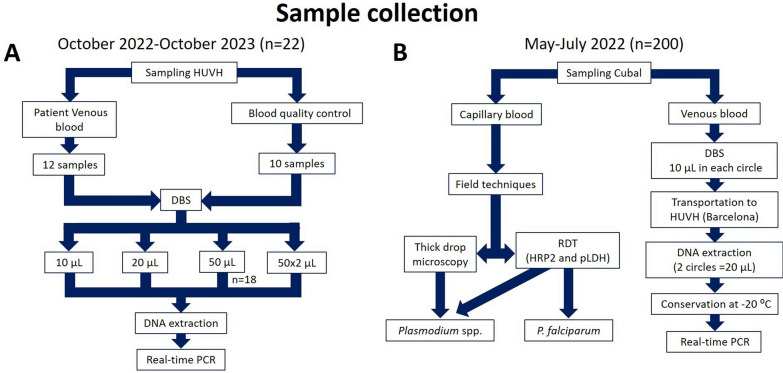

## Background

Malaria remains a global health concern, being especially devastating in resource-limited endemic settings located in tropical and subtropical regions [1]. The World Health Organization (WHO) reported approximately 249 million cases and 608,000 deaths from this parasitic disease in 2022 [2]. During this period, the African continent accounted for 93.6% of cases and 95.4% of deaths globally, with the sub-Saharan region being the most affected [2]. Angola is a malaria-endemic country in this region, harboring 3.4% of malaria cases and 3.2% of malaria deaths worldwide, making it the fifth most prevalent and seventh most deadly country in the world [2]. Cubal is a rural municipality in the province of Benguela (Angola) where malaria transmission has been described as stable mesoendemic [3]. In this type of setting, field surveys and research studies for active surveillance are considered useful tools for estimating the prevalence of malaria in the population, as they can identify transmission foci established by asymptomatic carriers and infections with low parasitemia [4–6].

The microscopic examination of blood smears has long been the technique of choice for determining the prevalence of malaria in epidemiological studies [7–9]. Currently, this technique also remains the gold standard for the diagnosis of this disease, allowing parasite density quantification and the identification of the *Plasmodium* species. However, sensitivity varies based on the observer's experience, and its detection limit is around 50-100 parasites/µl [10]. Moreover, often it cannot detect infections with low parasite densities [11] or differentiate mixed infections caused by two or more *Plasmodium* species [12]. Recently, rapid diagnostic tests (RDTs) have been implemented for field surveys. These tests are a simple rapid method that detects specific antigens of *Plasmodium* spp. and/or *Plasmodium falciparum*, such as panmalaric [lactate dehydrogenase (pLDH), and aldolase] or histidine-rich protein 2 (*Pf*HRP2), respectively. However, RDTs cannot quantify the parasite load or accurately identify non-*P. falciparum* species. Additionally, deletions in the *pfhrp2* gene have been reported in *P. falciparum* populations in various geographical areas, which can lead to a false negative result [13–16]. Some studies have reported that a significant number of *Plasmodium* spp. infections are missed by RDTs because their parasite density is below the detection limit of the test [17].

Nowadays, the emergence of molecular techniques, such as real-time polymerase chain reaction (PCR), has opened a new path in malaria diagnosis due to its numerous advantages. The high sensitivity, low detection limit (< 1 parasite/µl), possibility of quantifying parasite density and capacity to identify *Plasmodium* species and mixed infections are the main advantages; however, the high cost of the equipment and reagents used for real-time PCR has prevented its implementation in resource-limited settings [18–20].

One of the challenges in performing PCR in settings where resources are limited is the correct extraction of DNA from whole blood and/or storage conditions during sample transport to a reference center. Nevertheless, the dried blood spot (DBS) method for the conservation of blood samples is widely used [21]. DBS sampling consists of placing drops of capillary blood, obtained by finger prick or venous blood collected by venipuncture, on filter papers that can be stored, preserved and transported at room temperature (RT) [22]. The DNA extracted from DBS is of sufficient quality to be used in PCR reactions, typing and sequencing techniques [23].

The objective of this study was to evaluate the usefulness of DBS as a method of preserving and transporting blood samples from a resource-limited environment, such as Cubal (Angola), to a reference center for the diagnosis of malaria by real-time PCR.

## Methods

**Fig. 1:**
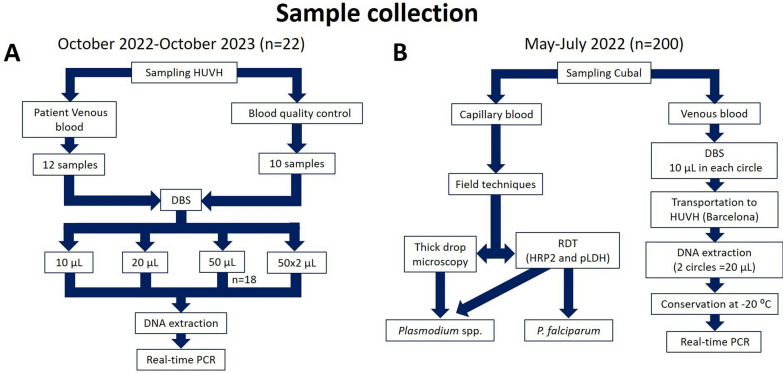
Flowchart established for the sample collection and diagnostic techniques used. **A** Schematic of the sampling carried out between October 2022 and October 2023 at the Vall d'Hebron University Hospital (HUVH, Barcelona, Spain) is shown. **B** Details of the procedure followed in the sampling carried out between May and July 2022 in Cubal

Two independent studies were conducted for related purposes (Fig. 1): to evaluate (i) the performance of real-time PCR for malaria diagnosis using DBS sampling, conducted at the Hospital Universitario Vall d’Hebron (HUVH, Barcelona, Spain), and (ii) the usefulness of DBS as a method for collecting and transporting samples without cold storage, conducted at the Hospital Nossa Senhora da Paz (HNSP, Cubal, Angola).

### Study population and sample collection

#### Performance of real-time PCR for malaria from DBS at HUVH

A prospective study was carried out at HUVH between October 2022 and October 2023. A total of 22 blood samples were included: 12 whole blood samples positive for *Plasmodium* spp. by real-time PCR from migrant patients from sub-Saharan countries who came to the HUVH for diagnosis of malaria and 10 quality control samples from QCMD 2023 *Plasmodium* spp. (Malaria) EQA Pilot Study (Quality Control for Molecular Diagnostics, Glasgow, UK). These control samples simulate human blood infected (*n* = 9) and not infected (*n* = 1) with *Plasmodium* spp. Different volumes (10 µl, 20 µl and 50 µl) of blood and quality control samples were used to obtain DBS samples on Whatman 903^™^ filter paper cards (GE Healthcare Life Sciences, Cardiff, UK). DBS samples were dried overnight at RT until DNA extraction for real-time PCR analysis (Fig. 1A).

#### Usefulness of DBS for malaria diagnosis by real-time PCR in Cubal, Angola

A cross-sectional study was conducted between May and July 2022 involving 200 samples from patients attending the HNSP for suspected malaria. Inclusion criteria were all patients presenting to HNSP with febrile syndrome. Recruitment was performed as described by Febrer-Sendra et al. [24]. Two types of samples were collected from each participant following the clinical protocol established by the HNSP: (i) a capillary blood sample obtained by finger prick was used for field diagnostic techniques (microscopy and RDT); (ii) a whole blood sample was obtained by venipuncture, from which 10 µl was inoculated in each circle of Whatman 903^™^ filter paper cards (GE Healthcare Life Sciences, Cardiff, UK). The young age and nutritional status of some of the participants, added to the other clinical tests performed with the same blood collection, meant that the DBS collection would be from venous blood and that the inoculated volume of blood in each circle of the filter paper could not exceed 10 µl. DBS samples were completely dried for 24 h at RT and stored at 4 °C until shipment to the HUVH; during shipment, the DBS samples were kept at RT. Once at destination, DBS samples were stored at − 20 °C until further molecular analysis. These storage conditions were established to preserve the highest possible quality of parasite DNA as it took several weeks before they were analyzed [25] (Fig. 1B).

### Field diagnostic techniques: microscopy and RDT

For microscopic examination, a thick blood smear was prepared from each capillary blood sample and stained with 10% Giemsa for 15 min. An experienced microscopist examined all slides at 100× magnification with immersion oil. For each smear, the presence or absence of *Plasmodium* spp. infection was determined, and parasite density was quantified according to the number of parasites per 100 leukocytes, assuming a quantity of 8000 leukocytes/µl of blood. A slide was determined as negative if no *Plasmodium* spp. parasites were observed after counting 500 leukocytes [24]. Three groups were established, according to the intensity of infection, as follows: low intensity (< 800 parasites/µl), medium intensity (800–4000 parasites/µl) and high intensity (> 4000 parasites/µl) [26]. The RDT used in this study was the STANDARD^™^ Q Malaria Pf/Pan Ag test (SD Biosensor, Republic of Korea). This RDT is a membrane-based immunochromatography for the qualitative detection of *P. falciparum*-specific *Pf*HRP2 and *Plasmodium* spp. (*P. falciparum*, *P. vivax*, *P. ovale* and *P. malariae*)-specific pLDH. The detection limit is 163 parasites/µl. The diagnosis of *Plasmodium* spp. infection by at least one of these two techniques was used as a reference method when determining the performance of real-time PCR from DBSs collected at HSNP.

### DNA extraction

DNA extraction from both DBS and whole blood samples was performed with the automated eMAG™ system (BioMérieux SA, Marcy-l'Étoile, France), according to the manufacturer’s instructions; both types of samples were eluted into 110 µl elution buffer.

For DBSs collected at HUVH, one circle inoculated with 10 µl, 20 µl and 50 µl of whole blood or two circles with 50 µl of whole blood were placed in individual 2-ml NUCLISENS^®^ lysis buffer tubes. For DBSs collected at HNSP, two circles infected with 10 µl of whole blood each were punched and placed into individual lysis buffer tubes (20 µl total volume of DBS per sample). Then, DBSs were incubated with continuous mixing at RT for 30 min. The filter paper debris was removed with sterile tweezers, and a rapid centrifugation was performed before starting the isolation program. For whole blood samples, a volume of 200 µl was used for DNA extraction. The eluates were stored at – 20 °C until further analysis by real-time PCR.

### Real-time PCR

All real-time PCR reactions were performed on the CFX96 Touch real-time PCR detection system (Bio-Rad, Hercules, CA). First, a real-time PCR for *Plasmodium* spp. detection (hereafter, generic PCR) was performed using the RealStar® Malaria PCR Kit 1.0 (Altona Diagnostics, Hamburg, Germany), following the manufacturer's instructions (limit of detection: 1.27; CI 0.57–5.42 copies/µl eluate). Samples that tested positive in the generic PCR were subjected to a second real-time PCR for identification of the *Plasmodium* species (hereafter, specific PCR) using the RealStar^®^ Malaria Screen & Type PCR Kit 1.0 (Altona Diagnostics, Hamburg, Germany) [limits of detection: *P. falciparum* = 0.80 (CI = 0.44–2.45), *P. vivax* = 0.73 (CI = 0.46–1.62), *P. malariae* = 0.36 (CI = 0.24–0.74), *P. ovale* = 1.46 (CI = 0.89–3.28) and *P. knowlesi* = 2.35 (CI = 1.37- 5.55) copies/µl of eluate].

A positive control (*Plasmodium* spp.-specific or species-specific DNA, obtained from DNA extraction from the blood of malaria patients attending HUVH, whose infection was confirmed by microscopy and real-time PCR) and a negative control (nuclease-free water), were included in each run. Samples were considered positive for *Plasmodium* DNA when the threshold cycle (Ct) for the *Plasmodium* target was < 40 and the internal control amplified effectively.

Although real-time PCR used in this study is not an absolute but a relative quantitative method, Ct values were used as an indirect estimate of parasite density [27, 28].

### Statistical analysis

Qualitative variables are expressed as percentages and absolute frequencies. Quantitative variables are described as mean or median and standard deviation (SD) or interquartile range (IQR) according to the normality of the data distribution.

Statistical analyses were performed using the R-UCA package for Windows (version R-UCA-3.3.1.exe). The statistical tests used were as follows: (i) single factor ANOVA and Tukey's test for pairwise comparisons to determine whether there were significant differences between real-time PCR from DBSs or whole blood; this test was also applied in the analysis according to infection intensity groups to detect any differences between real-time PCR Ct values from DBSs collected in Cubal; (ii) the Chi-square test of independence was used to determine the dependence between field diagnostic and real-time PCR results; (iii) the Kruskal-Wallis test followed by the Wilcoxon test was used to test for significant differences in parasite density determined by microscopy between the different groups established according to the intensity of infection; (iv) Spearman's correlation test was used to determine the correlation between real-time PCR Ct values from DBSs collected at Cubal and parasite density determined by microscopy. In all cases, the significance level was set at *P* < 0.05.

The differences in Ct values between DBS and blood samples (ΔCt) using real-time PCR for the study conducted at HUVH were calculated as follows:

$$\Delta Ct=\frac{\sum {(Ct}_{DBS}- {Ct}_{whole\, blood})}{n}$$

where ${Ct}_{DBS}$ is the Ct obtained from a particular DBS volume for a sample, ${Ct}_{whole blood}$ is the Ct obtained from whole blood of the same sample, and $n$ is the total number of samples analyzed for the same DBS volume.

For real-time PCR performed on the samples from the study conducted at HNSP, the following diagnostic performance parameters were calculated using microscopy and/or RDT results as a reference: sensitivity, specificity, positive predictive value (PPV) and negative predictive value (NPV). To establish the level of concordance of real-time PCR with reference results, Cohen's kappa coefficient was calculated and interpreted as follows: slight concordance (0–0.20), fair concordance (0.21–0.40), moderate concordance (0.41–0.60), substantial concordance (0.61–0.80) and almost perfect concordance (0.81–1.00).

## Results

### Performance of real-time PCR for malaria from DBS

#### Real-time PCR for the detection of *Plasmodium* spp. DNA

Real-time PCR from whole blood samples was positive in 100% (21/21) of *Plasmodium* spp.-infected samples and negative in the one uninfected sample. The number of positive samples from DBS varied according to the volume of blood inoculated: 76.19% (16/21) for 10 µl, 85.71% (18/21) for 20 µl, 88.24% (15/17) for 50 µl and 85.71% (18/21) for 100 µl. Notably, for four samples, it was not feasible to fill one circle of the filter paper with 50 µl. All samples with Ct > 34 in real-time PCR performed from whole blood were negative for all DBS volumes. The only sample that was PCR negative from whole blood was also negative for all DBS volumes. See Table 1.

**Table 1:** Detection and identification of *Plasmodium* species by real-time PCR from whole blood and DBS

	PCR result	Whole blood N (%)	DBS			
			10 µl N (%)	20 µl N (%)	50 µl N (%)^a^	100 µl N (%)
Generic PCR	*Plasmodium* spp.	21/21 (100)	16/21 (76.19)	18/21 (85.71)	15/17 (88.24)	18/21 (85.71)
Specific PCR^b^	*P. falciparum*	17/21 (80.95)	10/16 (62.50)	13/18 (72.22)	11/15 (73.33)	14/18 (77.78)
	*P. malariae*	3/21 (14.29)	2/16 (12.50)	2/18 (11.11)	2/15 (13.33)	2/18 (11.11)
	*P. falciparum* + *P. ovale*	1/21 (4.76)	0/16 (0)	0/18 (0)	0/15 (0)	0/18 (0)
	Undetermined	0/21 (0)	4/16 (25)	3/18 (16.67)	2/15 (13.33)	2/18 (11.11)

^a^In four samples, it was not possible to fill the filter paper circle with 50 µl

^b^Percentages are calculated according to the number of positive samples for *Plasmodium* spp.

**Fig. 2:**
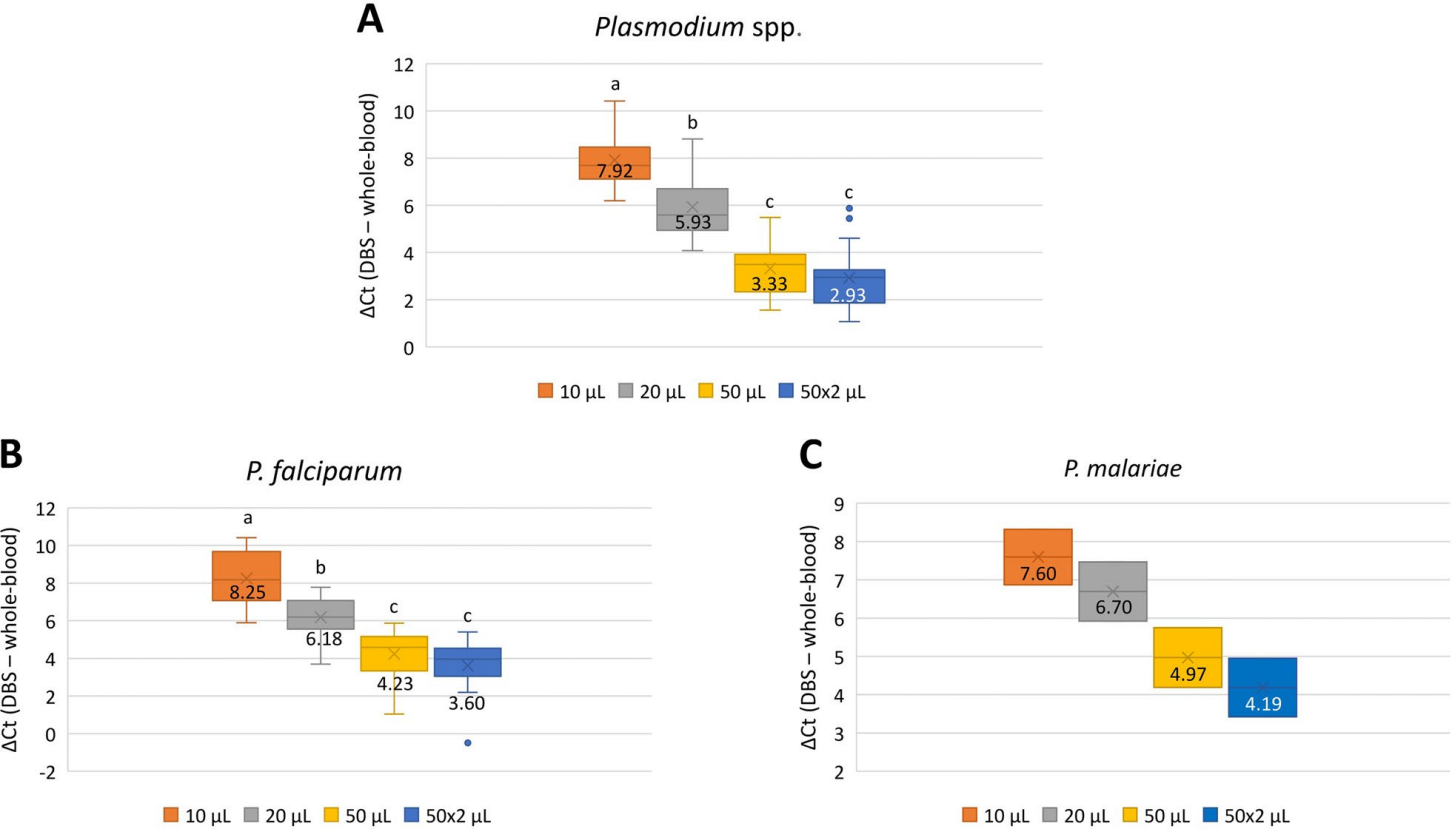
Yield loss (ΔCt) detection of real-time PCR performed on DNA extracted from DBS inoculated with different volumes of blood (10 µl, 20 µl, 50 µl and 100 µl) compared to extraction from whole blood (200 µl). The crosses inside the box and numbers indicate the mean value of the ΔCt. Letters indicate groups according to significant differences between mean ΔCt values, so that values with different letters (a, b or c) present statistically significant differences between them (*P* < 0.01). **A** ΔCt values of generic real-time PCR, **B** ΔCt values of *Plasmodium falciparum*-specific PCR and **C** ΔCt values of *Plasmodium malariae*-specific PCR. Significant differences were not analyzed for *P. malariae* as only two samples were positive

The yield loss of real-time PCR from the different DBS volumes used compared to whole blood (represented as ΔCt) is shown in Fig. 2. The highest loss was with the 10 µl DBS volume, with a difference of 7.92 Ct. The ΔCt for each DBS volume presented significant differences between them in all cases (*P* < 0.001), except between the 50 µl and 100 µl DBS groups (*P* > 0.05).

#### Real-time PCR for the identification of *Plasmodium* species

According to the PCR results from whole blood, 80.95% (17/21) of samples corresponded to *P. falciparum*, 14.29% (3/21) to *P. malariae,* and 4.76% (1/21) to mixed infection by *P. falciparum* and *P. ovale*. When a higher DBS volume was analyzed, *Plasmodium* species could be identified in a greater number of samples. The mixed infection sample could not be identified by PCR using any of the DBS volumes because the generic PCR for DBS was negative; therefore, the specific PCR was not performed. See Table 1.

ΔCt was observed in specific real-time PCR when using DBSs with whole blood (Fig. 2). For *P. falciparum,*, a higher ΔCt was observed using 10 µl of DBS compared with *P. malariae* detection. For *P. falciparum*, mean ΔCt values for each of the DBS volumes presented significant differences (*P* < 0.01), except between the 50 µl and 100 µl DBS groups (*P* > 0.05).

### Usefulness of DBS for malaria diagnosis by real-time PCR in Cubal, Angola

#### Real-time PCR performance

Field diagnosis (microscopy and/or RDT) determined that 51.5% (103/200) of participants were infected with *Plasmodium* spp. compared to 50% (100/200) detected by real-time PCR from DNA extracted from DBSs (*P* < 0.001). Microscopic examination detected 33.5% (67/200) positive and RDT 49% (98/200). The performance parameters (sensitivity, specificity, PPV, NPV and kappa coefficient) of real-time PCR from DBSs using field diagnosis as the reference are shown in Table 2.

**Table 2:** Performance of real-time PCR from DBS using field diagnosis (microscopy and RDT) as the reference

	Sensitivity (CI 95%)	Specificity (CI 95%)	PPV^a^ (CI 95%)	NPV^a^ (CI 95%)	Kappa (CI 95%)
Real-time PCR DBS	77.67 (68.19–85.04)	79.38 (69.72–86.66)	80.00 (70.57–87.08)	77.00 (67.31–84.58)	0.57 (0.46–0.68)

^a^PPV: positive predictive value, NPV: negative predictive value

**Fig. 3:**
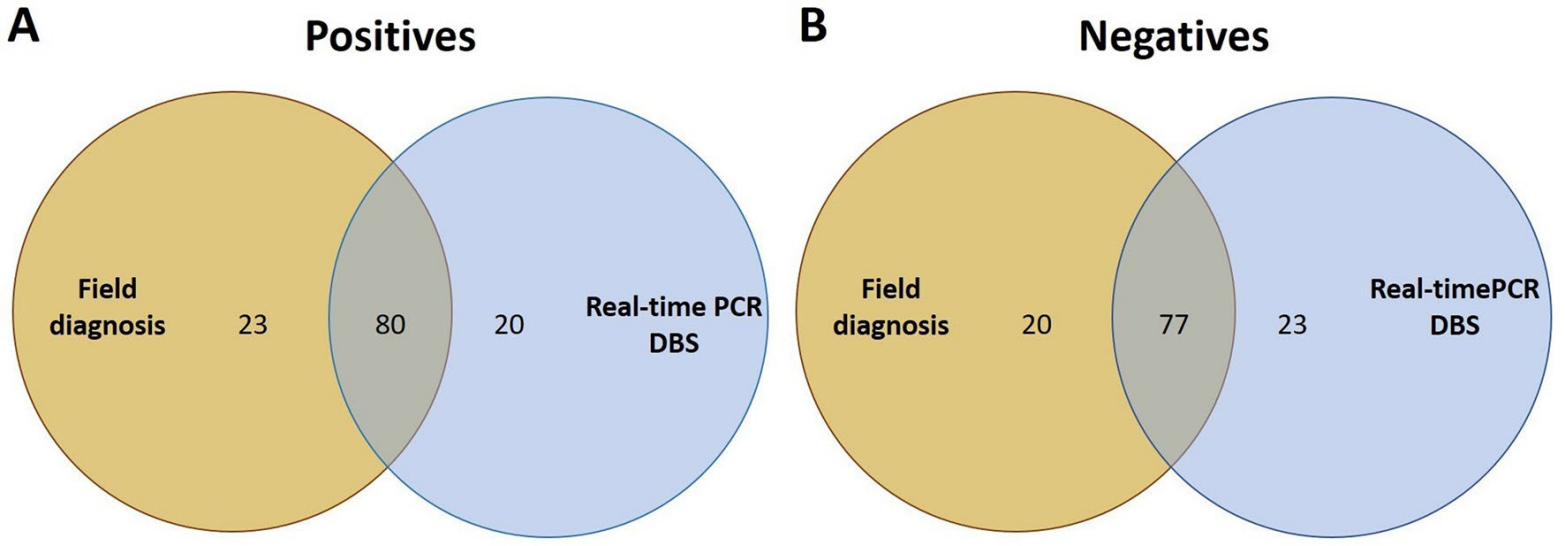
Venn diagrams for the comparison between real-time PCR from DBS and field diagnosis. **A** Distribution of positive and **B** negative samples for *Plasmodium* spp. infection with each technique

A comparison between the results of field diagnosis of malaria and diagnosis by real-time PCR is shown in Fig. 3. Of the total number of samples analyzed, 61.50% (123/200) were positive by field diagnosis and/or real-time PCR. Field diagnosis detected more positive cases than real-time PCR (23 *vs.* 20, respectively). Of these samples, only 34.78% (8/23) were microscopy-positive, and only two were not low-intensity infections (1103 and 5866 parasites/µl); the other discordant samples were only detected by RDT.

The mean Ct value obtained by real-time PCR was 30.76 (SD = 5.96). The median parasite densities obtained from microscopic examination were 6508 (IQR = 1223.5–19,731.5) parasites/µl. In addition, there was a significant correlation between real-time PCR Ct values and the parasite density quantification established by microscopy (*P* < 0.001, r = − 0.88). When groups were established based on the intensity of infection, it was found that as the intensity decreased, the mean real-time PCR Ct value increased significantly (*P* < 0.01) in each of the groups (Table 3).

**Table 3:** Intensity of infection established by microscopy and its relationship to real-time PCR from DBS samples collected in Cubal (*n* = 59)

Infection intensity	Infections	Microscopy	Real-time PCR
	N (%)	Parasite density (parasites/µl)^a^	Ct^b^
High	31 (52.54)	19315 (9819.50–77,973)a^c^	23.70 (2.14)a^c^
Moderate	14 (23.73)	1920 (1255.25–2523.75)b^c^	28.66 (2.77)b^c^
Low	14 (23.73)	424 (252.50–666.75)c^c^	32.35 (3.31)c^c^

^a^Results are expressed as median and IQR

^b^Results are expressed as mean and SD

^c^Letters indicate statistically significant differences between infection intensity groups for each technique (*P* < 0.05)

#### *Plasmodium* species identification by real-time PCR

**Fig. 4:**
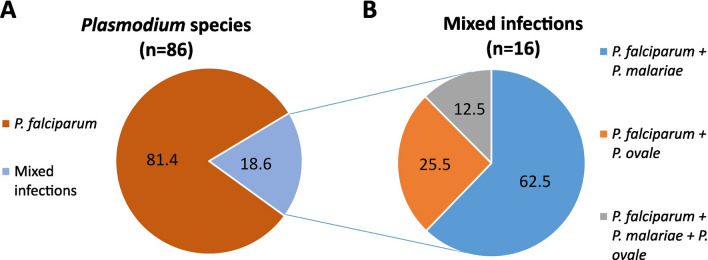
Molecular identification of *Plasmodium* species by real-time PCR from DBS collected in Cubal. **A** Percentages of the prevalence of *Plasmodium* species identified and of mixed infections are shown with respect to the total number of samples in which the species could be determined (*n* = 86). **B** The percentages of prevalence of the different mixed infections caused by two or more *Plasmodium* species are plotted (*n* = 16)

Of the samples positive for *Plasmodium* spp. DNA by real-time PCR, the species could not be determined in 14% (14/100) of the cases due to low sample parasitemia (first real-time PCR Ct values > 36.3). The most prevalent species was *P. falciparum*, present in 100% (86/86) of infections, followed by *P. malariae* in 13.95% (12/86) and *P. ovale* in 6.98% (6/86) of cases. *Plasmodium falciparum* was detected as a single species infection in 81.40% (70/86) and as a mixed infection in 18.60% (16/86) (Fig. 4A). Of the mixed infections, *P. falciparum* and *P. malariae* coinfection accounted for 62.5% (10/16), *P. falciparum* and *P. ovale* 25% (4/16), and triple coinfections with these three species were identified in 12.5% (2/16) of the cases (Fig. 4B).

## Discussion

The use of highly sensitive diagnostic methods, e.g. molecular techniques, is fundamental for accurately reporting the true prevalence of malaria in the population and interrupting transmission; however, the implementation of these techniques in rural settings is limited [21]. One potential approach would be to collect and transport the samples to a reference laboratory, but this presents certain challenges. In this sense, DBS sampling offers an efficient and applicable solution in resource-limited settings for the collection, conservation, and transport of whole blood samples [23]. The combination of molecular techniques together with the DBS method has been used in numerous research studies [15, 29–34].

Concerning the study of malaria real-time PCR performance from DBS samples at HUVH, a higher agreement between whole blood and DBS samples was observed in PCR results and species identification when larger volumes of whole blood were used for DBS (100 µl). However, of the 21 PCR-positive whole blood samples, 13.6% (3/21) of the DBS samples returned a negative result, even when using the DBS volume that gave the best results (100 µl). Our results agree with other published studies, with DBS PCR for 4% [22], 13.3% [35] and 27% [36] of infections detected from whole blood. The sensitivity of PCR from DNA extracted from DBS has been described to vary for different reasons: the blood volume used, type of filter paper [22], storage conditions (time, temperature and humidity) [25], extraction method used [37] and PCR kit chosen [22, 38]. Similarly, there appears to be an inherent yield loss when performing extraction of genetic material from DBS compared to whole blood. This might be due to incomplete recovery of the total volume of blood inoculated onto the filter paper during the pre-extraction step, possibly depending on the dilution factor set in the sample recovery.

Moreover, we found that DBS volume influences the sensitivity of real-time PCR. One possible explanation is that when performing nucleic acid extraction with larger blood volumes, the number of parasites and consequently the number of DNA molecules will be higher. In our study, the 50- and 100µl volumes resulted in a smaller loss of sensitivity. However, further investigations with a larger number of low parasitemia samples are necessary to determine whether a 100µl volume is better than a 50µl volume. Nevertheless, blood volume is usually a limitation when collecting samples in the field [36], and 50 µl could be sufficient for molecular studies of malaria in resource-limited settings. On the other hand, we made an important finding: when whole blood samples had a Ct value > 34, the corresponding DBS sample recorded a lack of amplification in real-time PCR, leading us to believe that infections with very low parasite densities may be missed with this technique.

In our study, all *Plasmodium* species identified were concordant between whole blood and DBS; however, in a small number of samples, identification was not possible. Differences have been observed in the performance of PCR from DBS depending on the *Plasmodium* species. Three independently conducted studies concluded that a higher proportion of infections were missed with *P. vivax* compared with *P. falciparum* when using DBS [22, 36, 39]. One potential explanation for this phenomenon is the lower parasitemia observed in infections caused by *P. vivax* compared to the high parasite densities frequently seen in *P. falciparum* infections [40]. Unfortunately, we did not identify any cases of *P. vivax*. However, we observed that the detection performance with *P. malariae* was lower than that of *P. falciparum* when 50- and 100µl DBSs were used.

Upon reviewing real-time PCR Ct values, we observed that whole blood samples with a Ct > 35 were those in which the *Plasmodium* species could not be identified. This led us to believe that the lower sensitivity of the specific PCR used in species identification compared with the generic PCR that detects *Plasmodium* spp. was the reason we could not identify these species from the DBS samples [28]. In addition, the only mixed infection found was also missed when using DBSs. Nonetheless, this was due to the low parasitemia of that sample as *Plasmodium* spp. infection was also not detected from DBSs.

Focusing on the field study, real-time PCR determined fewer infections than field diagnosis using both microscopy and RDT. This may be explained by the possibility of false-positive RDT results, as it is common in high transmission settings for this test to detect the *Pf*HRP2 antigen in the blood of a patient who has recently had malaria but was not infected at the time of testing [41]. However, PCR detected a higher proportion of positive samples compared with using microscopy or RDT independently. Our results agree with other studies conducted in Equatorial Guinea and the Democratic Republic of Congo, where PCR performed on DNA extracted from DBSs identified a higher number of positive samples than microscopy and RDT [31, 42].

Concerning the samples positive by field diagnosis and negative by real-time PCR, the possibility of false-positive RDT results due to the persistence of antigen after past infection, and the loss of PCR performance with DBS in low parasitemia samples may be the cause of these discrepancies. However, the failure of PCR to detect two samples with medium- and high-intensity infections remains unexplained. In contrast, a positive result with real-time PCR and a negative one for field diagnosis may be due to the higher sensitivity of the PCR technique, as demonstrated in previous studies [31, 42, 43].

In another recent study also conducted in Cubal, real-time PCR from DNA extracted in situ determined that 39.5% of the participants were infected with *Plasmodium* spp. [28] compared with 50% detected from DBSs in this study. In addition, in the present study, the sensitivity of real-time PCR regarding field diagnosis was considerably higher (77.67%) compared with PCR of DNA extracted in situ (64.08%) [28]. This led us to think that, during the transport of the DNA eluates, the genetic material of *Plasmodium* spp. underwent a strong degradation due to inadequate preservation conditions. In this sense, using DBS for sample transport is highly advantageous since it does not require the maintenance of a cold chain and allows the effective preservation of parasite DNA for detection by real-time PCR. Despite the loss of PCR sensitivity when performed on DBSs compared with whole blood, the advantages offered by DBS as a means of preservation and transportation of blood samples simplify the logistics of molecular studies in resource-limited settings, as is the case of Cubal.

Regarding quantification to estimation of infection intensity, we observed that real-time PCR from DBSs could detect one case of high-intensity and two cases of low-intensity infections that were missed in the study in which DNA extraction was carried out in situ [28]. In addition, mean Ct values were lower for all intensity groups in the case of DBS compared with in situ extracted DNA [28], especially in the high-intensity group. Therefore, real-time PCR Ct values from DBSs could be an indicator to estimate the intensity of infection, always considering the loss of sensitivity of this method compared with using whole blood.

To identify the *Plasmodium* species, PCR from DBS samples allows us to accurately identify these species and distinguish mixed and single infections [30, 47, 48]. As expected, real-time PCR from DBS was able to identify more species than PCR from in situ DNA extracts because of better DNA preservation [28]. In the previous in situ extraction study, two cases first categorized as mono-infections of *P. malariae* and *P. ovale* [28] turned out to be mixed infections of these species together with *P. falciparum* according to the results obtained from the DBS. Thus, DBS achieved a more accurate identification of the *Plasmodium* species and could detect mixed infections with greater precision than with in situ DNA extraction.

Some limitations were identified in this study that could affect the interpretation of the results. First, venous blood was used to collect DBSs instead of capillary blood. This decision was influenced by the young age and nutritional status of the participants, as well as the high number of diagnostic tests required, making venous sampling a more practical option in this setting. However, as a result, only two 10µl DBS circles could be collected per participant, which may have reduced the sensitivity of the real-time PCR performed on these samples. Second, the lower sensitivity of the species-specific PCR compared to the generic *Plasmodium* spp. DNA-targeted PCR limited our ability to identify *Plasmodium* species in cases with very low parasitemia. Finally, the persistence of antigens in the bloodstream may have contributed to false-positive results in RDTs, potentially leading to an overestimation of field diagnostic results. This overestimation may, in turn, have resulted in an underestimation of the sensitivity of real-time PCR from DBS when calculating its diagnostic performance.

## Conclusions

The volume of DBS used for DNA extraction is a determining factor in the efficacy of real-time PCR for *Plasmodium* DNA detection and species identification. In addition, DBS allows estimation of the intensity of infection based on real-time PCR Ct values. In light of the above findings, our recommendation is that DBS sampling be employed as the optimal methodology for the acquisition, transport and subsequent molecular analysis of samples in malaria research. However, it is important to note that microscopy and RDTs should continue to be employed in point-of-care diagnosis, as they facilitate immediate results that are vital for initiating treatment. In addition, the volume of blood collected, as well as the process of drying and transporting the samples, may pose problems that require attention, as they could affect the accuracy of the results. Consequently, further research is needed to determine optimal methodologies for using DBS in prevalence studies and in mapping residual malaria, which will be of vital importance for malaria elimination in endemic regions.

## Data Availability

The data supporting the conclusions of this article are included in this manuscript. The datasets used and/or analyzed during the current study are available from the corresponding author upon reasonable request.

## Abbreviations

Ct: Threshold cycle
DBS: Dried blood spot
ΔCt: Differences in Ct values between DBS and blood samples
HNSP: Hospital Nossa Senhora da Paz
HUVH: Hospital Universitario Vall d’Hebron
IQR: Interquartile range
NPV: Negative predictive value
pLDH: Panmalaric lactate dehydrogenase
PCR: Polymerase chain reaction
*Pf*HRP2: *Plasmodium falciparum* histidine-rich protein 2
PPV: Positive predictive value
RDT: Rapid diagnostic test
RT: Room temperature
SD: Standard deviation
WHO: World Health Organization
